# Guidelines for Field Surveys of the Quality of Medicines: A Proposal

**DOI:** 10.1371/journal.pmed.1000052

**Published:** 2009-03-24

**Authors:** Paul N Newton, Sue J Lee, Catherine Goodman, Facundo M Fernández, Shunmay Yeung, Souly Phanouvong, Harparkash Kaur, Abdinasir A Amin, Christopher J. M Whitty, Gilbert O Kokwaro, Niklas Lindegårdh, Patrick Lukulay, Lisa J White, Nicholas P. J Day, Michael D Green, Nicholas J White

## Abstract

Paul Newton and colleagues propose guidelines for conducting and reporting field surveys of the quality of medicines.

## Introduction

There has been relatively little apparent interest in the quality of medicines used to treat common life-threatening diseases despite the logical implication that poor-quality medicines will reduce the effectiveness of therapy and encourage drug resistance. Evidence suggests that a significant proportion of drugs consumed in the developing world are of poor quality [1–25]. Translating evidence on drug treatment outcomes into treatment policy is futile if the medicines actually used have substantially inferior efficacy compared with the medicines originally evaluated [7]. Poor-quality medicines are conventionally classified into three main categories: counterfeit, substandard, and degraded (Box 1).

**Box 1. Definitions**

**A counterfeit medicine** is “deliberately and fraudulently mislabelled with respect to identity and/or source. Counterfeiting may include products with the correct ingredients or with the wrong ingredients, without active ingredients, with insufficient active ingredient or with fake packaging” [11].

**Substandard medicines** “are genuine medicines produced by legitimate manufacturers that do not meet the quality specifications that the producer says they meet. For example, they may contain less (or more) active ingredient than written on the package. This may not be an intention to cheat, but may be due to problems with the manufacturing process” [12].

**Degraded medicines** may result from exposure of good-quality medicines to light, heat, and humidity. It can be difficult to distinguish degraded medicines from those that left the factory as substandard, but the distinction is important as the causes and remedies are different [13].

In addition, medicines used past their expiry date should also be regarded as poor quality—as they may also be degraded. However, there are very few data on what the expiry date for medicines used in the tropics should be, rather than the conventional three years. More investigation is required—three years may well be too short, or too long, for some medicines. If medicines can be used for longer after the conventional expiry date this would have important economic and drug safety benefits.

In many reports it is unclear whether a poor-quality medicine is counterfeit, substandard, or degraded.

The existing literature includes little discussion about the most appropriate sampling and reporting strategies for medicine quality surveys [2,7,15,16], and the majority of papers either have inadequate reporting of sampling methods and/or used “convenience” sampling, which is potentially flawed by bias. Depending on whether the medicine collectors, consciously or subconsciously, prefer to find poor-quality medicines (e.g., if it might result in publications or funding) or not (e.g., if it might cause embarrassment), they may overestimate or underestimate, respectively, the prevalence of outlets selling poor-quality medicines. Convenience sampling may lead investigators to sample more geographically accessible outlets, which may be unrepresentative of those used by patients.

**Summary Points**

- Despite evidence suggesting that substandard, counterfeit, or degraded medicines are major problems of global importance, there are few reliable data describing their epidemiology.
- Poor-quality medicines particularly affect lower-income countries, where information and drug regulation enforcement is scant, but inadequate infrastructure, non-regulated drug outlets, and black market operations make drug quality surveys difficult.
- We reviewed previous work on the quality of medicines and how medicine quality studies have been reported. We discuss best sampling strategies and suggest a draft checklist of appropriate items to be addressed in future studies.
- More research on medicine quality monitoring methodologies is needed, together with a standardisation of medicine collection protocols. The objective of the guidelines presented here is to guide surveys of medicine quality and how they are reported, and to provide a template for further development.

This paper has two main aims. First, we discuss how medicine quality surveys can be conducted and how simple and efficient but statistically valid sampling techniques can be used to provide an estimate of the prevalence of outlets selling low-quality medicines. This discussion is based upon a literature review and consultation with experts in the field (five physicians, four chemists, three pharmacists, two statisticians, and two public health epidemiologists), involved in research on poor-quality medicines (Box 2: Methods). Second, we discuss how such studies may be reported and propose a checklist (Medicine Quality Assessment Reporting Guidelines [MEDQUARG]) to facilitate transparent, consistent, and accurate reporting, in the hope that robust evidence will assist in improving medicine quality. A fuller version of this discussion document is available in Text S1.

**Box 2. Methods**

We first searched the medical literature through PubMed, Google Scholar, and the World Health Organization Web site using the keywords “counterfeit”, substandard”, “fake”, “medicine quality”, and “drug quality” for information and guidance related to the conduct and reporting of medicines quality surveys. PNN, FMF, and MDG created a draft document summarising the literature, and PNN, SJL, LJW, and NJW contributed to the statistical section. We then undertook a consultation by circulating multiple sequential drafts (about six) to an additional ten people who had recently published on the subject. They were contacted by e-mail and asked if they would be able to contribute—none declined. PNN incorporated their comments into this consensus document, and all participated with the iterative process and agree with the document presented here. We also posted the draft document paper on the Enhancing the QUAlity and Transparency Of health Research (EQUATOR) network Web site [38] for four weeks to request comments by e-mail from a wider community and incorporated the response received.

## Strategies for Conducting and Reporting Medicine Quality Surveys

### 1. Sampling techniques.

Informed decisions on appropriate sampling size and strategies are currently very difficult as there are no published reliable estimates for the prevalence of poor-quality medicines or the proportion of outlets selling such medicines for any country. The sampling strategy will depend on the question being asked, such as “Are there medicines of poor quality in a particular geographical area?” or “Is the proportion of outlets selling poor-quality medicines above a pre-determined acceptable level, and/or what is the prevalence of poor-quality medicines in this geographical area?” The sampling unit(s) for analysis may be the outlets and/or the medicines sold from them. The distinction is important as, for example, an area may have one outlet selling 50% of the poor-quality medicine(s) bought in the region or ten outlets each selling 5% of the poor-quality medicines. Weighting may be required based on the number of treatments dispensed per outlet, which could be derived from household surveys or sales volumes declared by the outlets. Surveys have usually estimated the proportion of poor-quality medicines collected in outlets [4,10,11,14,17–25] and not the proportion of shops selling poor-quality medicines. We suggest that both types of measures should be reported [18]. By using the proportion of medicine outlets selling poor-quality essential medicines as the unit of observation and a standardised randomised sampling procedure of sufficient sample size, it will be possible to map distribution and allow comparisons through time.

There has been no discussion as to what proportion of outlets selling poor-quality medicines should be regarded as unacceptable. Ideally there should be zero tolerance for poor-quality medicines as even a 1% prevalence of such medicines for potentially fatal diseases, such as malaria, tuberculosis, and HIV, is disastrous. However, as 30% of World Health Organization member states are said to have either “no medicines regulation or a capacity that hardly functions” [12], it is extremely unlikely that these medicines regulatory agencies (MRAs) will be able to reduce the prevalence of poor-quality medicines to less than 1%. It is currently recommended that national malaria treatment policy should be changed when about 10% of patients fail treatment [26]. It is therefore logical that strenuous efforts should be made to improve the quality of antimalarials available such that the proportion of outlets selling ineffective antimalarial medicines is less than 10%. The threshold values (see below) that determine what is an unacceptable proportion of outlets selling poor-quality medicines would presumably be higher in countries with good medicines regulation and should rise as MRAs develop capacity.

Convenience surveys, in which collectors sample medicines without specific guidance as to which outlets to sample, have been the predominant technique used. They are simple and relatively inexpensive and are the only sampling technique that does not require complete lists of outlets in defined areas, which may be difficult to obtain, especially for unlicensed or mobile outlets. However, they are inherently prone to biases. The results are dependent on the collector's choice of outlets, and prevalence estimates can have no reliable associated measure of confidence. Changes in the prevalence of poor-quality medicines, and outlets selling them, through time derived from convenience sampling cannot be interpreted reliably as changes may simply represent sampling artefact. Nevertheless convenience surveys may provide the initial signal of a problem (analogous to case reports of adverse effects to a drug), and may provide evidence to support legal action in police and MRA investigations. If convenience sampling does indicate a drug quality problem, we suggest that more objective methods be used in subsequent surveys. If the sampling suggests that drug quality is good, this may be a false negative result.

A more objective technique is random sampling, which with sufficient sample sizes will give reliable estimates of the prevalence of outlets selling poor-quality medicines and their distribution in the defined area [27,28]. However, there are only three published studies in which random sampling has been used [22–25]. A random survey can be stratified by geographical, trade, and socioeconomic variables. Comparisons with subsequent estimates are valid and will allow the evaluation of interventions. The disadvantages of random sampling are the large sample sizes needed and the associated costs. It is important that a true randomisation procedure is used, such as from formal random number tables or using simple statistical software.

Lot quality assurance sampling (LQAS), to determine whether the prevalence of outlets selling poor-quality medicines exceeds a certain threshold, may be the most economical first step before deciding whether a randomised survey is required [29–31]. LQAS was developed to determine whether a batch (lot) of goods met the desired specifications without having to inspect the entire lot (Box 3: Example [32]). Thus, the sample size in LQAS is defined as the number of “units” that are selected from each lot, and the outcome is either “acceptable” or “unacceptable”. Setting the level of risk taken by not inspecting each item, the investigator is able to accept or reject an entire lot after inspecting a randomly selected sample. The sample size in LQAS is based on defined threshold values that classify good and bad outcomes and the probability of error that the investigators are willing to tolerate. The first step is to determine the upper and lower threshold values. For example, an area in which 20% or more of the outlets sell poor-quality medicines may be considered a “bad” situation since the risk of buying poor-quality medicines will be high, whereas 5% or less may be considered a “good” situation since the risk of buying poor-quality medicines will be lower. Next, acceptable probabilities of error must be specified; i.e., the risk of accepting a “bad” lot (“consumer risk”, Type I [alpha] error) and the risk of not accepting a “good” lot (“provider risk”, Type II [beta] error). The former is often set to 0.05. This means that if the null hypothesis (the defective goods proportion is less than the specified value) is true, there is a 5% chance that an unacceptable lot would be accepted. In general, the consumer risk is set lower than the provider risk. Once the threshold values and probabilities of error have been considered, a sample size and decision value can be obtained (see Box 3: Example). The decision value is the number of “defective” items that need to be found before a lot is considered unacceptable.

**Box 3: Example**

There is interest in determining the prevalence of outlets selling poor-quality co-artemether, the national first-line recommended treatment for malaria, on an island called San Serriffe [27].

**Random Sampling**

We can estimate a sample size assuming a prevalence of 50% (or *p* = 0.5). This choice of estimated prevalence will give us the most conservative (i.e., largest) sample size needed. To determine the actual prevalence of outlets selling counterfeits with a precision of 5% (below 0.05 × 2 = 0.1) with 95% confidence intervals (*z* = 1.96), we would need a random sample size (*n*) of ~390 (*n* = 4*p*(1-*p*)*z* ^2^/precision^2^ = 4 × 0.5(1-0.5 × (1.96)^2^/(0.1)^2^) [28, Table 6.1]. This means that purchases from 390 different outlets selling co-artemether would be required to obtain an objective estimate of the prevalence of those selling poor-quality co-artemether at one time point in one region.

**LQAS**

For LQAS sampling we set our upper threshold to 95% and the lower threshold to 80%. This means that it is acceptable for 95% of outlets selling artemether-lumefantrine (the unit) in one district (the lot) in San Serriffe to have good-quality medicines and unacceptable for less than 80% to have good-quality medicines. Then we set the Type I error to 0.05 (i.e., there is a 5 in 100 chance that a district with 80% or fewer of the outlets selling good-quality drugs will go undetected) and the Type II risk to 0.10 (i.e., there is a 10 in 100 chance that we will inappropriately direct resources to a district in which 95% or more of the outlets are in fact selling good-quality drugs). Our sample size would be 38 randomly selected outlets, and the district would be considered unacceptable if more than four outlets had poor-quality artemether-lumefantrine (calculated using SampleLQ [32]). In other words, the null hypothesis that the district has at least 80% of its outlets selling good artemether-lumefantrine would be rejected if more than four out of 38 outlets sold poor-quality artemether-lumefantrine.

LQAS still requires random (i.e., unbiased) sampling and has the disadvantage that it does not estimate an exact prevalence, but the advantage of requiring smaller sample sizes. Moreover, sampling can stop once the number of outlets with poor-quality medicine is exceeded, greatly reducing sampling time and costs [30]. If the number of outlets with poor-quality medicines exceeds the predefined number, further investigation with a larger random sample could be performed to measure the prevalence of outlets selling poor-quality medicines, and to examine accurately longitudinal changes. LQAS is relatively easily carried out and has been shown to give accurate and useful information that is translatable into policy [29–31,33–35].

Sentinel site monitoring involves following the quality of medicines at a particular locality through time [15]. There is no consensus as to whether these sites should be chosen on the basis of potentially important variables such as rural versus urban and private versus public outlets, nor on sampling methodology. Although the power of sentinel site monitoring resides in following longitudinal changes in one place, it suffers from the disadvantage that shop owners will probably soon realise that they are being sampled and will change their behaviour accordingly, and thus will no longer be representative of the population.

### 2. Who should sample?

Reports often do not state who was responsible for sampling medicines and how the collectors were chosen, and thus the likelihood that sellers would realise that they were participating in a survey. If the seller knows or is concerned that his/her stock contains illegal or poor-quality medicines and that the buyer is potentially linked to the MRA, this will greatly influence what medicines are offered for sale [36]. However, if the outlet staff are anxious to avoid selling poor-quality medicines, open sampling with feedback would allow more data to be collected and allow direct improvement in the medicine supply. In the face of uncertainty as to the sellers' awareness, we suggest that mystery shoppers [37] are the appropriate collectors in most circumstances and that sampling be performed by nationals of the country concerned. They should use a scenario, stating, for example, that they are visiting from another part of the country and would like some medicines for disease X for reason Y for a stereotyped patient Z without stating or giving any indication that they are not a “normal” shopper.

### 3. What, when, and how much to sample?

Outlets vary greatly in type and may be classified according to the local drug law and number and training of staff. Public health considerations should be the main guides for which types of outlets and what medicines and where to sample. In resource-poor settings, medicines sampled should be those on the country's essential medicines list, emphasising the outlets most widely used. Surveys with the collection of a restricted number of samples per batch may result in errors—e.g., fake and genuine tablets of the antimalarial artesunate may have the same batch numbers [8]. As outlets may have more than one brand of a particular medicine available, decisions should be made before sampling as to which to request. If a selection has to be made, this should be done randomly (Sengaloundeth et al., unpublished data).

A problematic issue is the number of dosage units to sample. Thirty dosage units for a single tablet/capsule medicine of a lot number from each location have been recommended [16]. Such a sample size gives enough dosage units to determine identity and content of active ingredients, dissolution, and degradation. However, many outlets in the rural tropics do not have 30 dosage units per medicine, and a request for such a large quantity is likely to suggest that the buyer is not an ordinary shopper [10,36]. We therefore suggest a smaller sample size of dosage units. The collection of between five and ten units should allow assessment but may not be sufficient for legal purposes.

### 4. Ethical and legal aspects of sampling.

Whether ethical review or informed consent is necessary to sample medicines from those selling them has not been widely debated; if this issue is of concern, the survey should be discussed with the appropriate ethical committee(s) and the affected communities [37]. If poor-quality medicines are detected, we suggest that the investigators have a duty to report the results to the local MRA so that they can make their own legal investigations and the evidence can be used to improve national medicine quality.

### 5. Costs.

Medicine quality surveys can be expensive, mostly because of the costs of chemical analysis, and this has inhibited such work with the result that we have very little objective information. However, given the large expense of clinical trials and medicines and the enormous economic burden of life-threatening diseases, this lack of investment is a false economy. More investment in laboratory infrastructure and personnel training is needed. It has been argued that surveys with random selection of outlets are not necessary, too complicated, or too expensive. We suggest that they are vital and that the additional expense in comparison to the chemical analysis cost is small.

### 6. Reporting.

The MEDQUARG guidelines (Table 1) consist of a checklist of items that we propose should be included in reports of medicine quality. These are not an attempt to prescribe the reporting of such research in a rigid format [38] and will evolve as more information and experience in this field becomes available. Wherever possible publications describing medicine quality should provide manufacturer's names as stated on the packaging [39]. Care should be taken to avoid legal action by the stated manufacturer, and it is the responsibility of the authors to determine whether or not to take legal advice before publication. Suggestions made in this article do not constitute legal advice and may not be relied upon to replace legal advice. However, it is our opinion that the phrase “stated to be manufactured by...” can be used as a statement of fact and does not mean that the manufacturer stated on the packaging actually manufactured the product.

**Table 1:**
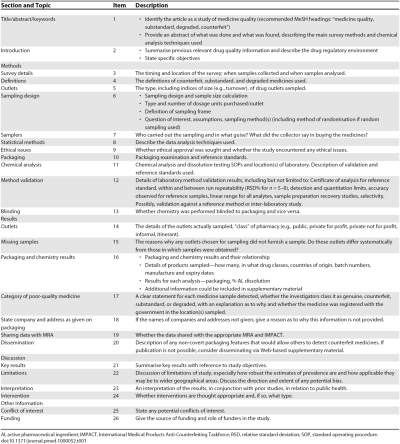
MEDQUARG Checklist of Items That We Suggest Should Be Addressed in Reports of Surveys of Medicine Quality

## Conclusions

Poor-quality medicines are a major impediment to improvements in public health. The quantity and quality of data available to those trying to improve the quality of the medicine supply for life-threatening diseases is woeful. We have discussed survey techniques to estimate the frequency of poor-quality medicines in geographical areas and have highlighted LQAS as a potentially accurate, relatively inexpensive, and useful screening tool for initial checking of whether the number of outlets selling good-quality medicines is acceptable. We also present a first draft of reporting guidelines, which we hope will be discussed and improved through posting of responses to this paper. The health of people living in developing countries is critically dependent upon the availability of good-quality medicines. We hope that this field will attract the interest and support it deserves, and that the recommendations made here will evolve.

## Supporting Information

Text S1 Longer version of the article (190 KB PDF).

## Abbreviations

LQAS: lot quality assurance sampling
MRA: medicines regulatory agency
MEDQUARG: Medicine Quality Assessment Reporting Guidelines
