# Supplementary material for: Guidelines for Field Surveys of the Quality of Medicines: A Proposal
Source: PLoS Med. 2009 Mar 24;6(3):e1000052. doi: 10.1371/journal.pmed.1000052 (PMC2659710; doi:10.1371/journal.pmed.1000052)
Supplement: Supplementary file 1 [file pmed.1000052.sd001.pdf]

Guidelines for Field Surveys of the Quality of Medicines: A Proposal

Paul N Newton[1,2,3], Sue J Lee[2,4], Catherine Goodman[5,6], Facundo M. Fernández[7], Shunmay Yeung[2,6], Souly Phanouvong[8], Harparkash Kaur[3], Abdinasir A Amin[9], Christopher JM Whitty[3], Gilbert O Kokwaro[5], Niklas Lindegårdh[2,4], Patrick Lukulay[8], Lisa J White[2,4], Nicholas PJ Day[1,2,4], Michael D Green[10], Nicholas J White[1,2,4]

1. Wellcome Trust-Mahosot Hospital-Oxford University Tropical Medicine Research Collaboration, Microbiology Laboratory, Mahosot Hospital, Vientiane, Lao PDR

2. Centre for Clinical Vaccinology and Tropical Medicine, University of Oxford, Churchill Hospital, Oxford, UK

3. Department of Infectious and Tropical Diseases, London School of Hygiene and Tropical Medicine, University of London, United Kingdom

4. Faculty of Tropical Medicine, Mahidol University, 420/6 Rajvithi Rd., Bangkok 10400, Thailand

5. KEMRI/Wellcome Trust Programme, Nairobi, Kenya

6. Health Economics and Financing Programme, Health Policy Unit, London School of Hygiene and Tropical Medicine, University of London, United Kingdom

7. School of Chemistry and Biochemistry. Georgia Institute of Technology, Atlanta, GA., USA

8. Drug Quality and Information Program, International Technical Alliances Program, United States Pharmacopeia, Rockville, MD, USA

9. Management Sciences for Health, Strengthening Pharmaceutical Systems Program. P.O Box 8700-00100, Nairobi, Kenya

10. Division of Parasitic Diseases, National Center for Infectious Diseases, Centers for Disease Control and Prevention, Atlanta, GA 30333, USA

Reprints or correspondence: Paul Newton. Wellcome Trust- Mahosot Hospital-Oxford Tropical Medicine Research Collaboration, Microbiology Laboratory, Mahosot Hospital, Vientiane, Lao PDR. Phone & Fax (856) 21 242168. E-mail: paul@tropmedres.ac

Abstract 258 words, Text 6,734 words

Abstract

Background

Public health research has tended to avoid studying the quality of essential medicines. Despite evidence suggesting that substandard, counterfeit, or degraded medicines are major problems of global importance, there are few reliable data describing their epidemiology, or their effects on health and drug resistance. Poor quality medicines particularly affect lower income countries, where information is scant and enforcement of drug regulations is often weak. Inadequate infrastructure, non-regulated drug outlets, and black market operations make statistically-sound drug quality surveys difficult. There are few guidelines available on how surveys should be conducted or reported.

Methods and Findings

We reviewed previous work on the quality of medicines and discuss the advantages and disadvantages of convenience sampling, random sampling, sentinel site sampling and lot quality assurance sampling (LQAS). Convenience sampling is prone to bias but may provide evidence for police action when a problem with medicine quality is suspected. We suggest that random-sampling LQAS is probably the most efficient and accurate initial sampling procedure with subsequent larger scale formal random sampling if a significant problem with medicine quality is revealed. We also reviewed how medicine quality studies have been reported and suggest a draft checklist of appropriate items to be addressed in future studies (Medicine Quality Assessment Reporting Guidelines (MEDQUARG).

Conclusions

More research on medicine quality monitoring methodologies and on the efficacy of interventions is needed, together with standardisation of medicine collection protocols. The objective of the consensus guidelines presented here is to guide surveys of medicine quality and how they are reported, and to provide a template for further development.

Keywords: Fake, counterfeit, substandard, medicines, sampling, sample size, drug quality, survey guidelines, lot quality assurance sampling, random sampling

Introduction

There is considerable recent interest in determining the burden of diseases, such as malaria, in tropical countries where most of the world's infant and child deaths occur [1]. There has also been a marked increase in the number of clinical trials conducted in tropical countries to determine the most efficacious and appropriate local treatments [2], their cost-effectiveness [3], and the factors determining the gap between efficacy and effectiveness [4]. Surprisingly, there has been relatively little apparent interest in the quality of medicines used to treat common life-threatening diseases despite the logical implication that poor-quality medicines will reduce the effectiveness of therapy and encourage resistance. There is evidence that a significant proportion of drugs consumed in the developing world are of poor quality, often with no active ingredient [5-12]. There has been little research into poor quality medicines, with the exception of a few surveys [19,27,30,33,35], of medicine quality and evaluations of rapid, inexpensive tests [13,14],

with and only one assessment of an intervention to improve medicine quality [15]. Such research and monitoring work appears relatively difficult to find funding for and to publish despite its obvious and immediate relevance. Medicine quality is an essential translational link between epidemiology/clinical trial research and improved public health [10]. Translating evidence on drug treatment outcomes into treatment policy is futile if the medicines actually used are substantially inferior in terms of efficacy or toxicity compared with the medicines originally evaluated. Poor-quality medicines are conventionally classified into three main categories: counterfeit, substandard and degraded (Box 1 [16-18]). In many reports, it is unclear whether a poor quality medicine is counterfeit, substandard, or degraded. For example, a recent paper did not distinguish substandard and counterfeit medicines (in the sense of the WHO definition) because 'neither is clinically suitable' [19]. But the distinctions are extremely important from a preventive standpoint as the causes and solutions are different. Even if the amount of active ingredient in a pharmaceutical preparation is correct, this is insufficient information to determine accurately whether a medicine is genuine. Detailed inspection of the packaging is required. For solid oral dosage forms, incorrect excipients or incorrect quantities of the correct excipients, different particle size, crystalline or amorphous state of the active ingredient, and poor formulation procedures can all contribute to poor dissolution resulting in lower bioavailability and a substandard medicine (see Appendix 1).

There is surprisingly little objective information on the prevalence of poor quality medicines, especially data that distinguish counterfeit from substandard. As a

consequence, there has been considerable confusion and uncertainty about these estimates, which are usually “best guesses” based on anecdotal information [10,20]. There has also been little discussion as to the most appropriate sampling and reporting strategies [5,10,21,22]. An aim of this paper is to stimulate debate so that consensus can be reached and more objective and comparable studies conducted in the future. To date, the majority of papers on medicine quality either have given inadequate reporting of sampling methods and/or used ‘convenience’ sampling, which is potentially flawed by bias. Depending on whether the medicine collectors, consciously or subconsciously prefer to find (e.g. if it might result in a publication or additional funding), or not find poor quality medicines (e.g. if it might cause embarrassment, panic, danger, or is part of a larger criminal or political agenda), they may underestimate or overestimate, respectively, the prevalence of outlets selling poor-quality medicines. Convenience sampling may lead investigators to sample more geographically accessible locations, such as in towns, or easier to identify outlets, such as licensed pharmacies, which may be unrepresentative of those used by patients. It would be inconceivable today to estimate the prevalence of, say, hypertension in a community by measuring the blood pressure of the people one happened to meet in the local market–but that is what most medicine quality sampling strategies have done [7,11,16,19,23-27].

The sampling strategy will depend on the question being asked. Examples of common questions include:

1. Are there medicines of poor quality in a particular geographical area?

2. Is the proportion of outlets selling poor quality medicines above a pre-determined acceptable level and/or what is the prevalence of poor quality medicines in this geographical area?
3. What are the proportions of different types of poor quality medicines–counterfeit, substandard or degraded?
4. What are the relative proportions of different types of outlets selling poor quality medicines of different types in a given geographical or pharmaceutical supply system?
5. What are the risk indicators of poor quality medicines in terms of packaging and chemical characteristics, geography, stated origin, and batch numbers?
6. Has the proportion of outlets selling poor quality medicines and the prevalence of poor quality medicines changed over time or after an intervention?
7. What are the supply chains by which poor quality medicines are distributed and the market segments they serve?

This paper has two main aims (for Methods see Box 2). First, we discuss how medicine quality surveys can be conducted and how simple and efficient but statistically valid sampling techniques can be used to provide an estimate of the prevalence of outlets selling low-quality medicines. This is a requirement for objective and valid comparisons and to test the effectiveness of interventions. Second, we discuss and suggest a consensus statement (Medicine Quality Assessment Reporting Guidelines (MEDQUARG)), similar to that for clinical trials (CONSORT [29]) to facilitate transparent, consistent, and accurate reporting, in the hope that robust evidence will assist in improving medicine

quality. This discussion is based upon a literature review and consultation with experts in the field (five physicians, four chemists, three pharmacists, two statisticians, and two public health epidemiologists), involved in research on poor-quality medicines (Box 2: Methods).

Strategies

1. Sampling techniques

Informed decisions on appropriate sampling size and strategies are currently very difficult as there are no published reliable estimates for the prevalence of poor-quality medicines or the proportion of outlets selling such medicines for any country. There are also very few data on the geographical distribution of poor quality medicines in relation to population density, borders, disease distribution, public versus private health facilities, trade routes, and socioeconomic status. For example, it is not known anywhere whether poor-quality medicines are more common in rural areas, where patients are often more disadvantaged and medicine outlets receive less attention from medicines regulatory authorities (MRA) or police, than in urban areas. Do substandard and fake medicines differ in their geographical distribution and market segment? For example, relatively rich people may be more likely to receive a fake artemisinin derivative for malaria, than poorer people, as fake artemisinins are still many times more expensive than genuine or substandard clinically inferior conventional antimalarials. Are substandard or counterfeit medicines more common in the private than in the public sector? Firmer recommendations on sampling methods will have to await such information.

All sampling techniques, except convenience sampling, require a sampling frame from which the sample can be drawn. This sampling frame must be representative of the population in which you want to be able to generalise your results. Therefore lists of the sampling locations, such as licensed and unlicensed outlets in geographically defined areas are required. These may be difficult to obtain, especially for the unlicensed outlets, but are essential information for such surveys. Many outlets are mobile. It may not be possible to map the “territory” of itinerant medicine sellers but an estimate of what proportion of the total number of sellers are itinerant could be used to generate a sensitivity analysis.

The sampling method will also need to take into account the level in the drug supply chain at which poor quality medicines enter the market. The sampling process in countries where there are only a few distributors from which all outlets obtain medicines will be very different from the process in countries with multiple independent distributors selling directly to small outlets.

The sampling unit for analysis may be the outlets and/or the medicines sold from them. The distinction is important as, for example, an area may have one outlet selling 50% of the poor quality medicine(s) bought in the region or 10 outlets each selling 5% of the poor quality medicines. Weighting may be required based on the number of treatments dispensed per outlet, which could be derived from household surveys or sales volumes declared by the outlets. However, such medicine usage information may not be available, especially for unlicensed outlets. Although it seems likely that the size of the outlet

would be roughly proportional to dispensing this may not be correct. For example, small peripheral pharmacies or shops may have relatively high throughput for drugs, such as antimalarials, which are used predominantly by the rural population. These factors will determine which indicators of medicine quality are reported, for example:

* proportion of medicine X products which are counterfeit/substandard/degraded in area A
* proportion of outlets selling any counterfeit/substandard/degraded medicines of classes A-Z in area A
* proportion of medicine X products which are counterfeit/substandard/degraded in area A weighted by medicine X use/sold per outlet.

Surveys have usually estimated the proportion of either a wide [15,30,31] or narrow [7,19,23] range of poor quality medicines in outlets and not the proportion of shops selling poor quality medicines. We suggest that both types of measures should be reported [24]. By using the proportion of medicine outlets selling poor-quality essential medicines as the unit of observation and a standardized randomized sampling procedure of sufficient sample size, it will be possible to map distribution and allow comparisons through time. Such a procedure will require knowledge of the expected underlying prevalence to calculate the sample size. If this knowledge is unavailable the worst-case scenario must be assumed, necessitating larger sample size.

There has been no discussion as to what proportion of medicines or outlets selling poor-quality medicines should be regarded as unacceptable. Ideally there should be zero-tolerance for poor quality medicines, as even a 1% prevalence of such medicines for potentially fatal diseases, such as malaria, tuberculosis and HIV, is disastrous. However, as 30% of World Health Organization member states are said to have either 'no medicines regulation or a capacity that hardly functions' [17], it is extremely unlikely that these medicine regulatory agencies (MRAs) will be able to reduce the prevalence of poor-quality medicines to <1%. It is currently recommended that national malaria treatment policy should be changed when ~10% of patients fail treatment [32]. For antimalarial medicines it is therefore logical that strenuous efforts should be made to improve the quality of antimalarials available such that the proportion of outlets selling ineffective antimalarial medicines is <10%. The thresholds values (see below) that determine what is an unacceptable proportion of outlets selling poor quality medicines would presumably be higher in countries with good medicines regulation and should rise as MRAs develop capacity.

A variety of sampling techniques can be employed, individually or in combination, to estimate the prevalence of poor quality medicines. Each possibility is discussed below.

A. Convenience surveys

Convenience surveys, in which samples are collected without specific guidance on which outlets to sample, have been the predominant technique used. As the name implies, convenience surveys are simple and relatively inexpensive and are the only sampling

technique that does not require complete lists of outlets in defined areas, which may be difficult to obtain, especially for unlicensed or mobile outlets. However, they are inherently prone to biases. The results of convenience sampling are crucially dependent on the collector's choice of outlets and prevalence estimates derived cannot be generalised to other areas, even within the same country. Changes in the prevalence of poor quality medicines, and outlets selling them, through time derived from convenience sampling cannot be interpreted reliably as changes may simply represent sampling artefact. Nevertheless convenience surveys may provide the initial signal of a problem (analogous to case reports of adverse effects to a drug) and may be useful during routine post-marketing medicines quality monitoring, particularly when doubts are raised about the quality of a specific medicine in a particular area. They may be very useful in police and MRA investigations and provide evidence to support legal action. Whenever convenience sampling is used there should be an attempt to report on how the sites were identified and the proportion of the outlets this represents. If convenience sampling does indicate a drug quality problem, we suggest that more objective methods be used in subsequent surveys. If convenience surveys do not demonstrate a problem one should bear in mind that this may be a false negative result.

B. Random sampling

A more objective technique than convenience sampling, which will improve the generalisability of results, is random sampling. However, there are only three published studiesof drug quality in which random sampling has been used [30,33-35]. With a

sufficient sample size, random sampling will give reliable estimates of the prevalence of outlets selling poor quality medicines and their distribution in a defined area (see Example). A random survey can use stratified sampling to adjust for potential differences in geographical, trade and socioeconomic variables, such as rural *versus* urban, private *versus* public and one geographic area *versus* another. Sampling proportional to population size (or number of medicine outlets) will be more efficient compared to simple random sampling. It is important that a true randomisation procedure is used, such as from formal random number tables or using simple statistical software.

Comparisons with subsequent estimates are valid and will allow the evaluation of interventions. An example of this technique is described in a recent stratified random sampling of the quality of anti-infective medicines in the Lao PDR [36]. The random sampling of medicine outlets was accomplished by the collectors in the field making numbered lists of outlets and telephoning a central location giving the total number of outlets found. The central location staff then used random number tables to tell the field team which outlet numbers to sample. The disadvantages of random sampling are the large sample sizes needed and the costs in labour and funds.

C. Lot Quality Assurance Sampling

Lot quality assurance sampling (LQAS) can be used to determine whether the prevalence of outlets selling poor-quality medicines exceeds a certain threshold, and may be the most economical first step before deciding whether a formal randomised survey is required. LQAS was developed in the 1920s to assess the quality of industrially produced goods

[39-41]. The impetus was to determine whether a batch, or lot, of goods met the desired specifications without having to inspect the entire lot. Thus, the ‘sample size’ in LQAS is defined as the number of ‘units’ that are selected from each lot and the only outcome in this kind of sampling is ‘acceptable’ or ‘unacceptable’. Setting the level of risk taken by not inspecting each and every item enables the investigator to accept or reject an entire lot after inspecting a randomly selected sample of items. The sample size in LQAS is based on defined threshold values that classify good and bad outcomes and the probability of error that the investigators are willing to tolerate. The first step is to decide upon these upper and lower threshold values (see Example). For example, an area in which 10% or more of the outlets sell poor-quality medicines may be considered a ‘bad’ situation since the risk of buying poor-quality medicines will be high, whereas 5% or less may be considered a ‘good’ situation since the risk of buying poor quality medicines will be lower.

Next, acceptable probabilities of error must be specified; the risk of accepting a ‘bad’ lot (‘consumer risk’) and the risk of not accepting a ‘good’ lot (‘provider risk’). These risks are commonly referred to as Type I (alpha) and Type II (beta) errors, respectively. The former is often set to 0.05. This means that if the null hypothesis (the defective goods proportion is less than the specified value) is true, there is a 5% chance that an unacceptable lot would be accepted. In general, the risk of accepting an unacceptable lot is set lower than the risk of classifying an acceptable lot as unacceptable. Once the threshold values and probabilities of error have been considered, a sample size and decision value can be obtained. The decision value is the number of ‘defective’ items

that need to be found before a lot is considered unacceptable. LQAS still requires random (i.e. unbiased) sampling and has the disadvantage that it does not estimate an exact prevalence, but the advantage of requiring smaller sample sizes (see Example). If the number of outlets with poor quality medicines exceeds the predefined number, further investigation with a larger random sample could be performed to measure the prevalence of outlets selling poor quality medicines. Intervention could be instituted, and further LQAS, or preferably larger scale random sampling, arranged to assess the impact of the intervention. Moreover, sampling can stop once the number of outlets with poor quality medicine is exceeded, greatly reducing sampling time and costs [40]. Double-sampling plans can also be used to allow further economies–if the results from a small sample size are extreme, there is evidence of a significant problem with medicine quality, and the survey can stop. If the results of the preliminary sampling are equivocal a second larger sample can be chosen and conclusions based on the combined sample [40]. As LQAS will only provide a binary result, formal random sampling will be required to examine accurately longitudinal changes in the prevalence of poor quality medicines.

LQAS has been used widely to monitor health programmes, such as assessing immunization coverage and the reading of TB smears [38-40,43,44]. There are many LQAS guidelines (e.g. [45]) and much experience in the use of the technique for diverse public health problems in countries with poor medicine quality. We suggest that LQAS may be an appropriate initial sampling strategy for detecting those areas with poor medicine quality. Research suggests that it is easily taught and carried out and gives accurate and useful information that is easily translatable into policy [43-44].

Comparisons of cost, diagnostic validity and field applicability with other sampling strategies have been performed [41] and we suggest that similar studies be conducted to assess the utility and cost of LQAS in medicine quality surveys.

D. Sentinel site monitoring

Sentinel site monitoring, in which the quality of medicines in a particular locality are followed longitudinally, has been used [21]. There is no consensus as to whether these sites should be chosen on the basis of potentially important variables such as rural *versus* urban and private *versus* public outlets, nor on sampling methodology (i.e. convenience or random samples or LQAS). Although the power of sentinel site monitoring resides in allowing longitudinal changes to be followed in one place, it suffers from the disadvantage that shop owners will probably soon realise that they are being sampled and, will change their behaviour accordingly and thus no longer be representative of the population.

2. Who should sample?

Reports often do not state who was responsible for sampling medicines and how the collectors were chosen, and thus the likelihood that sellers would realize that they were participating in a survey. Procedures as to who should do the sampling will be dependent on the regulatory status of the medicine(s) in question, whether the seller knows whether he/she is selling poor-quality medicines and understands the health, legal, and ethical implications. Many outlets in countries with weak medicines regulation sell unlicensed

medicines, which even if of good quality, may make outlet staff suspicious and anxious about investigations. Anecdotal reports suggest varying levels of knowledge among drug sellers regarding the quality of their wares [7,46], but there are no objective data as far as we are aware. If the seller knows or is concerned that his/her stock contains illegal or poor quality medicines and that the buyer is potentially linked to the MRA, this will greatly influence what medicines are offered for sale [31]. However, if the outlet staffs are anxious to avoid poor quality medicines, open sampling with feedback would allow more data to be collected on poor quality medicines and their risk factors and direct improvement in the medicine supply. Open sampling may be the only possible method in some circumstances, such as if samples are collected where people are seen first by clinicians.

In the face of uncertainty as to the sellers' awareness we suggest that mystery shoppers [47] are the appropriate collectors in most circumstances and that sampling be performed by nationals of the country concerned. It may not be safe for people living in the same community to act as purchasers. The shopper should not give any indication that they are not a 'normal' shopper' and should dress and behave appropriately without signs or speech suggesting that they are come from an urban elite if the sampling is conducted in rural areas. They should use a scenario, stating, for example, that they are visiting from another part of the country and would like some medicines for disease X for reason Y for a stereotyped patient Z, without stating or giving any indication that they are not a 'normal' shopper. If many dosage units of a large number of medicines are requested the seller may become suspicious and the medicines collected may not reflect what is

actually available at those outlets [31]. No studies appear to have been performed to examine what medicines different ‘types’ of collectors are sold.

An additional concern is that in many resource-poor countries the medicine market is heavily segmented with different markets for people of different spending power and ethnicity. For example, the least poor may go to pharmacies or private clinics, whilst the poorest go to grocery shops or street peddlers and people of middle income may go to hospitals. Even within a single outlet there will often be several different brands of the same product aimed at different market segments, with for example, a 'local' brand (least expensive), a non-local and non-European (middling expensive) and a 'European' brand (expensive). Therefore, what the mystery shopper will collect will depend partly on how wealthy the shopkeeper thinks the shopper is. This emphasises the need for more information on medicines use and dispensing practices and the importance of sampling guided by the volume consumed rather than that displayed.

3. What, when and how much to sample?

Outlets vary greatly in type and may be classified by MRAs according to the countries’ drug laws and by mobility, number and training of staff. Whether sampling should stratify the outlets in this way for analysis should be discussed during the survey planning. To allow comparison between countries with different outlet classification terminologies, outlets could be classed as public (Government), private for profit (e.g.

private pharmacies, supermarkets, grocery shops), private not for profit (e.g. mission hospitals, NGOs) and informal (e.g. kiosks, street vendors). Locations can be classified, by incidence/prevalence of the disease for which the medicines are produced for or by degree of urbanization.

Which medicines should be sampled, and in which area, will depend on what is already known or suspected. Public health considerations and the potential consequences of poor quality medicines should be the main guides for what medicines to sample. In resource-poor settings, medicines sampled should be those on the country's essential medicine list emphasising the outlets most widely used. We have found no information on seasonal changes in poor quality medicine availability. For example, if fake antimalarials are more available during the malarious season the timing of surveys will be important.

There may be more than one brand or batch of medicine of a particular type per outlet. The usual aim of medicine quality surveys is to determine what patients are likely to use rather than to monitor the quality of different batches of medicine. The ideal procedure would be to buy samples of all brands and batch numbers available per outlet, but that would be very difficult, expensive and unusual and obviously alert the vendor! Surveys with the collection of a restricted number of samples per batch may result in errors–e.g. fake and genuine tablets of the antimalarial artesunate may have the same batch numbers [11]. As outlets may have more than one brand of a particular medicine available, decisions should be made before sampling as to which to request and, if a selection has to be made, this should be done by random selection to avoid bias from seller or

investigator. In a country-wide survey of artesunate quality in Laos, if multiple brands of artesunate were available, the mystery shopper consulted a hand written note of random numbers for different numbers of brands, which stated which medicine to buy, counting from the left when the brands were lined up by the seller [36]. For example, if an outlet had 4 brands displayed and the random number table gave numbers 2 and 4, the collector would select the second and fourth, counting from the left.

A problematic and unresolved issue is the number of dosage units to sample. The United States Pharmacopeia (USP) [21] recommends 30 dosage units for a single tablet/capsule medicine of the same lot number from each location. Such a sample size gives enough dosage units to determine identity and accurate estimates of content of active ingredients, dissolution and degradation. However, many outlets in the rural tropics do not have 30 dosage units of a particular medicine, especially expensive brands, and so a request for such a large quantity is likely to suggest to the outlet owner that the buyer is not an ordinary shopper [15]. One alternative would be to sample key medicines at the level of the wholesaler where requests for large quantities would be routine. The USP guidelines on dosage unit sampling were designed for analyses that would withstand examination in a court of law, such as would be needed by a MRA to press legal charges. The results of the surveys as discussed here are unlikely to be used in courts, but would suggest to MRAs problems which should be investigated using their correct legal procedures. We therefore suggest a smaller sample size of dosage units. The collection of between 5 and 10 units should allow assessment of identity, content of active ingredients, dissolution and degradation. Although they are not routinely available yet, new non-destructive high

throughput mass spectrometry, Raman and IR spectroscopy techniques can perform assays for identity and active ingredient content, followed by assays for disintegration and dissolution, allowing smaller sample sizes. A standard reporting form should be used to record details such as collection date and site, cost and type, location, type and size of outlet. Samples should be stored in airtight plastic bags or plastic containers with unique identification codes permanently marked. The general accepted practice has been to store most sampled medicines in a dark, dry, well-ventilated room at temperatures of 15^0C to 25^0C, unless otherwise indicated on the label. Certain heat-sensitive medicines should be kept in between 4^0C and 8^0C [22].

4. Ethical and legal aspects of sampling

Most of the authors of this review do not believe that ethical review approval or informed consent is necessary to sample medicines from those selling them. We have found no statement that researchers have considered this issue and suggest that medicine outlets have, by selling medicines, assumed a duty of care and a responsibility to allow inspection. Once it has been suspected that substandard or fake medicines are being sold, there is an ethical responsibility to confirm or refute the suspicion as soon as possible, as ignoring the issue would be unethical, as would ignoring an identified source of infectious disease or poisoning. However, not all may agree [47] and we suggest that, if this issue is of concern, the survey should be discussed with the appropriate ethical committee(s) and the affected communities. Whether ethical approval is needed will probably depend more on what questions are asked rather than the sampling per se. If

poor-quality medicines are detected we suggest that the investigators have a duty to report the results to the local MRA so that they can make their own legal investigations and that the evidence can be used to improve national medicine quality. It would be unethical for a research project to provide erroneous information, as a result of poor sampling design or chemical analysis, which may either falsely discredit a good medicine supply or falsely reassure MRAs or patients that a poor quality medicine supply is good.

An additional neglected ethical problem is the potential difficulty of collectors buying all the stock of an essential medicine from a shop where there are no or few other suppliers within reach. Replacement of the sampled stock with quality assured medicines by the investigators, immediately after the survey, might be appropriate. If mystery shoppers are used this would require informing the outlets that they had been sampled after sampling has occurred. This may allow more information to be collected if the outlet staff cooperate with the investigators but may also increase the risk of disputes if they do not. Whether the MRA should be involved in the survey is potentially difficult but we argue that this is preferable. However, in some circumstances involving the MRA could greatly complicate the survey. For example, involving the Karnataka MRA in a survey during the period when corrupt officials were issuing false licenses [48] would have likely led to considerable complications and an inaccurate result. On the other hand such involvement may have led to exposure of corrupt practices before the Government of India discovered the problem. It is likely that most essential medicines consumed are not issued with a prescription, especially in impoverished areas. Depending on local pharmaceutical law, sampling where prescriptions are required may be more difficult, as prescriptions issued

to mystery shoppers could be regarded as counterfeit and illegal and prior discussion with the MRA would be essential.

Medicine sampling by academics is unlikely to lead to evidence that could be used in a court of law as the evidence is not normally collected using legally robust ‘chain of evidence’ procedures [49]. We are not aware of any academic research that has led directly to prosecutions. The recent investigation of the criminal epidemiology of fake artesunate provided non-legal evidence that prompted the Chinese Government to investigate and obtain locally appropriate evidence that could be used in a court of law [11].

5. Chemical Analysis

A. Chemical analysis

The paucity of quality assurance laboratories with appropriate managerial capacity, analytical capabilities and adequate resources for these investigations has also been a major hindrance to progress. There is no international quality assurance system, that we are aware of, that allows inter-laboratory comparisons of the quality of the drug analysis results (see Appendices 1-3). Crucially, there is remarkably little investment in building capacity, including that for chemical analysis, in the 30% of MRAs that have either no medicines regulation or a capacity that hardly functions [17]. Further problems include the difficulties of obtaining reference samples of packaging and active ingredients, often made a continent away. As with the sampling strategy, the chemical analysis strategy will

depend on the questions being asked. In the absence of budgetary constraints, the key outcome would be to determine accurately if the medicines sampled are counterfeit, substandard or degraded and what proportion of the active ingredient is present. For example:

1. If packaging suggests that samples are fake, does their chemical composition support this?
2. What is the chemical composition, including unexpected active ingredients, of the poor quality medicines sampled?
3. For medicines with the correct qualitative composition and packaging, are they substandard or degraded?
4. What are the forensic relationships between different fakes and what are the likely errors that led a medicine to be substandard?

The choice of chemical analysis methods should be determined before the study starts and will be driven by the question being asked, counterbalanced by the cost and availability of the analytical tools [50,51]. The different techniques available and their advantages and disadvantages are summarised in Appendix 2. The measures that should be considered include physical appearance, weight uniformity, dimensions, hardness, % active ingredient, disintegration, dissolution (Appendix 1) and, for liquid preparations, cloudiness, precipitates and microbiological contamination. As modern analytical methods often require highly trained personnel and are very expensive, an evaluation should be made prior to sampling as to whether the human resources are in place to carry out the analysis. Both counterfeit and substandard medicines may have no active

pharmaceutical ingredient, too little, too much or a wrong active ingredient. However, the data available suggest that most counterfeit medicines contain no or wrong active ingredients and substandard medicines contain too little or too much of the active ingredient [10]. The acceptable limits for a pharmaceutical preparation (often between ~90 and ~110% of the quantity stated on the label) are dependent on the medicine and are listed in reference pharmacopoeias [52-54]. Apart from formal high performance liquid chromatography (HPLC) and mass spectrometry (MS), innovative near-infrared (NIR) and Raman methods using hand held instruments are being evaluated [55,56]. They may allow rapid, inexpensive screening of medicines, through the packaging, once the portable instruments (which are expensive) have been bought. Although we are unaware of any economic evaluations, it is likely that these new techniques would be highly cost effective for MRAs lacking financial and human resources for large scale, quality-assured HPLC and MS laboratories. Other non-chemical forensic analyses, which may assist in determining likely sources of counterfeit medicines, include analysis of the pollen and other organic and inorganic remains derived from the environment in which the medicines were made [11]. The assessment of the prevalence of substandard medicines is confounded by the lack of information regarding stability of some medicines kept in inadequate transportation and storage conditions in tropical climates. There are no guidelines as to how substandard and degraded medicines can be distinguished and we suggest that genuine poor quality medicines should be regarded as substandard unless clear evidence for degradation is obtained. It is likely that future investigation of poor quality medicines by MS will help distinguish the two categories through identification of degradation products.

B. Packaging

The methods of packaging analysis (e.g. hand lens, UV light) and the comparator genuine examples should be described and, to avoid bias, the person performing packaging analysis should be blinded to the chemical analysis and vice versa. Detailed inspection should include examination of the packaging materials (packet, blisterpack, leaflet insert), the labelling of trade, brand and generic names, dosage form, strength, lot or batch number, name and address of manufacturer or distributor, manufacturing date, expiry date, quantity contained in the package unit, registration number, logo or hologram, the physical examination of the actual dosage forms for abnormalities. Errors such as spelling mistakes, batch numbers and expiry dates can provide clues when they differ from the genuine product [11]. Subtle differences in the morphology of holograms [11] and colour of packaging [57] can be detected using analysis of scanned packaging. Simple UV light bank note checkers can reveal covert features on the genuine product and their absence or copies on the fake [11]. Formal hologram analysis can reveal the type of holographic process used to manufacture counterfeit holograms and printing analysis to distinguish the printing methods used on the packaging [11,51]. Accessible web-based libraries of examples of genuine and counterfeit medicines may assist in the detection of counterfeits [11,58,59]. The differences should be made publicly accessible, allowing MRAs, pharmacists and patients to distinguish the genuine from the counterfeits, [11,24,59]. Covert features, which the counterfeiters have not detected, should remain confidential beyond the investigators and MRAs.

6. Costs

Medicine quality surveys can be expensive, in part because of the travelling and staff time required but mostly because of the costs of chemical analysis. The logistic difficulties and chemical analysis expense has inhibited such work, with the result that we have very little objective information. However, given the large expense of clinical trials and of medicines and the enormous economic burden of life-threatening diseases this lack of investment is a false economy. "Low-tech" field pass/fail methods and high-throughput laboratory screening methods are effective options for reducing the analysis cost per sample. More investment in laboratory infrastructure and personnel training is needed. It has been argued that surveys with random selection of outlets are not necessary, too complicated or too expensive. We suggest that they are vital and that the additional expense in comparison to the chemical analysis cost is small. In the stratified random sampling of medicine quality in Laos, the methodology, in comparison to convenience sampling of the same area, added a few hundreds of dollars to the cost due to the additional time of discussion and training, additional work in ensuring that lists of licensed and unlicensed outlets were up to date and increased travel to telephones to obtain the random number codes [36]. We suggest that a survey that will give robust estimates of the prevalence of outlets distributing poor quality medicine can be performed with only slightly increased costs and effort, in comparison to a convenience survey, and this is highly likely to be cost-effective. A risk assessment should be carried out in collaboration with the appropriate police forces and strict confidentiality observed for those participants thought to be at risk. Those involved in counterfeiting are by definition

criminal, often involved in organised transnational crime [60] and attempts have been made on the lives of people combating the problem [61].

7. **Reporting**

The draft MEDQUARG guidelines (Table 1) consist of a checklist of items that we propose should be included in reports of medicine quality. These are not an attempt to prescribe the reporting of such research in a rigid format [29] and will evolve as more information and experience in this field of research become available, and there are more discussions on the ethical and legal implications and statistical methodologies. It has been argued that secrecy within the pharmaceutical industry and governments has hampered progress in improving medicine quality [6]. Indeed, in 38% of reports recently reviewed [10], either the country of origin or trade name/company of the pharmaceutical was not given in the original publication. Whenever possible manufacturer's names, as stated on the packaging, should be reported [62]. Care should be taken to avoid legal action by the stated manufacturer and it is the responsibility of the authors to determine whether or not to take legal advice before publication. Suggestions made in this article do not constitute legal advice and may not be relied upon to replace legal advice. However it is our opinion that the phrase "stated to be manufactured by..." can be used as a statement of fact and does not mean that the manufacturer stated on the packaging actually manufactured the product. Most reports of poor quality medicines are currently not reported in the scientific literature but are held by pharmaceutical companies and MRAs or highlighted by journalists. The Western Pacific Region of WHO has pioneered a rapid alert system to disseminate information on poor quality medicines among MRAs which

could be expanded globally. There is also a great need for a web based system in which the public can check the correct packaging and tablet appearance of registered products and reports of poor quality medicines – with the information released when MRAs, and not pharmaceutical companies, decide that it is in the public interest [6].

8. Conclusions

Poor-quality medicines are a major impediment to improvements in public health. Despite much public and governmental concern with medicine quality in the 17th-early 20th centuries, since the formation of MRAs in the 20th century there has been surprisingly little research on the extent of the problem and on the effectiveness of interventions. The quantity and quality of data available to those trying to improve the quality of the medicine supply for life-threatening diseases is woeful. We have discussed survey techniques to estimate the frequency of poor-quality medicines in geographical areas and have highlighted random sampling LQAS as a potentially accurate, relatively inexpensive screening tool for initial checking of whether the number of outlets selling good-quality medicines is acceptable. We also present a first draft of reporting guidelines, which we hope that others interested in this subject will discuss and improve upon through posting of responses to this paper. The health of people living in developing countries is critically dependent upon the availability of medicines. Ensuring that essential medicines are of good quality is as important as ensuring that they are available. We hope that this field will attract the interest and support it deserves, and that the recommendations made here will evolve substantially.

Acknowledgements

We are very grateful to numerous colleagues for useful discussions and advice, especially Clive Ondari, Valerio Reggi, Budio Santoso and Eva-Marie Christophel of WPRO, Manila, Iveta Simera of EQUATOR, Kasia Stepniewska, Bonnie Cundill, Dallas Mildenhall, Aline Plançon, Sian Clarke, Frank Davidoff, Catherine Moyes and Carolyn McKee of the Legal Services Office, University of Oxford.

Funding

FMF thanks the World Health Organization Regional Office for the Western Pacific for funding support. PNN, FMF, HK, CJMW, MDG and NJW thank the Bill & Melinda Gates Foundation for support through the ACT Consortium. AAA is a Senior Program Associate at Management Sciences for Health working on the Strengthening Pharmaceutical Systems (SPS) Program (US Agency for International Development cooperative agreement number GHN-A-00-07-00002-00), based in Nairobi, Kenya. CG is a member of the Consortium for Research on Equitable Health Systems (CREHS), which is funded by the UK Department for International Development. SP and PHL thank the US Agency for International Development (USAID) for financial support through U.S. Pharmacopeia Drug Quality and Information Program. PNN, SL, SY, NL, LJW, NPJD and NJW are supported by the Wellcome Trust of Great Britain. The funders had no role in study design, data collection and analysis, decision to publish, or preparation of the manuscript.

Competing Interests

None of the authors have a competing interest

References

1. Dean T, Jamison DT, Breman JG, Measham AR, Alleyne G, et al. (2006) Disease Control Priorities in Developing Countries. Second edition. Washington & New York: Oxford University Press and The World Bank. 1394 p. Available: http://files.dcp2.org/pdf/DCP/DCP.pdf. Accessed 11 October 2008.

2. Myint HY, Tipmanee P, Nosten F, Day NP, Pukrittayakamee S, et al. (2004) A systematic overview of published antimalarial drug trials. Trans R Soc Trop Med Hyg 98: 73–81.

3. Arrow KJ, Panosian CB, Gelband H (2004) Saving lives, buying time: Economics of malaria drugs in an age of resistance. Washington (DC): Institute of Medicine of the National Academies. Available: http://www.nap.edu/books/0309092183/html/. Accessed 5 August 2007.

4. Amin AA, Hughes DA, Marsh V, Abuya TO, Kokwaro GO, et al. (2004) The difference between effectiveness and efficacy of antimalarial drugs in Kenya. Trop Med Int Hlth 9: 967-974.

5. Shakoor O, Taylor RB, Behrens RH (1997) Assessment of the incidence of substandard drugs in developing countries. Trop Med Int Hlth 2: 839–845.

6. Cockburn R, Newton PN, Agyarko EK, Akunyili D, White NJ (2005) The global threat of counterfeit drugs: why industry and governments must communicate the dangers. PLoS Medicine 2: e100.

7. Newton PN, Proux S, Green M, Smithuis F, Rozendaal J, et al. (2001) Fake artesunate in southeast Asia. Lancet 357: 1948-1950.

8. Newton PN, Rozendaal J, Green M, White NJ (2002) Murder by fake drugs – time for international action. British Medical Journal 324: 800-801.

9. Newton PN, McGready R, Fernández FM, Green MD, Sunjio M, et al. (2006) Manslaughter by fake artesunate in Asia –will Africa be next? PLoS Medicine 3: e197.

10. Newton PN, Green MD, Fernández FM, Day NJP, White NJ (2006) Counterfeit anti-infective medicines. Lancet Infectious Diseases 6: 602-613.

11. Newton PN, Fernández FM, Plançon A, Mildenhall DC, Green MD, et al. (2008) A forensic epidemiological investigation into the criminal fake artesunate trade: an international collaboration between police, scientists, and health workers. PLoS Medicine 5: e32.

12. Amin AA, Kokwaro GO (2007) Antimalarial drug quality in Africa. J Clin Pharm Ther 32: 429-440.

13. Green MD, Nettey H, Villalva Rojas O, Pamanivong C, Khounsaknalath L, et al. (2006) Use of refractometry and colorimetry as field methods to rapidly assess antimalarial drug quality. J Pharm Biomedical Anal 43: 105-110.

14. Green MD, Nettey H, Wirtz RA (2008) Determination of Oseltamivir Quality by Colorimetric and Liquid Chromatographic Methods. Emerg Infect Dis 14: 552-556.

15. Syhakhang L, Lundborg CS, Lindgren B, Tomson G (2004) The quality of drugs in private pharmacies in Lao PDR: a repeat study in 1997 and 1999. Pharm World Sci 26: 333-8.

16. Wondemagegnehu E (1999) Counterfeit and substandard drugs in Myanmar and Viet Nam. WHO Report. WHO/EDM/QSM/99.3. Geneva: WHO.

17. World Health Organisation (2005) Counterfeit and substandard drugs. Frequently asked questions. Available at: http://www.wpro.who.int/media_centre/fact_sheets/fs_20050506.htm. Accessed 27 February 2009.

18. Keoluangkhot V, Green M, Nyadong L, Fernández F, Mayxay M, Newton PN (2008) Impaired clinical response in a patient with uncomplicated falciparum malaria who received poor quality and underdosed intramuscular artemether. Am J Trop Med Hyg 78: 552-555.

19. Bate R, Coticelli P, Tren R, Attaran A (2008) Antimalarial drug quality in the most severely malarious parts of Africa - a six country study. PLoS One 3: e2132.

20. IMPACT (2006) Counterfeit Medicines: an update on estimates 15 November 2006. Available: www.who.int/medicines/services/counterfeit/impact/TheNewEstimatesCounterfeit.pdf. Accessed 26 January 2008.

21. Phanouvong S, Blum N, Smine A (2004) Guidelines for sampling of antimalarial drug samples in the USP DQI antimalarial drug quality monitoring project in Mekong sub-region countries. USP. Available: http://www.usp.org/pdf/EN/dqi/samplingGuidelines.pdf. Accessed 7 April 2008.

22. United States Pharmacopeia/DQI (2007) Ensuring the Quality of Medicines in Resource-Limited Countries: An Operational Guide. Rockville, MD: The United States Pharmacopeia Convention. Available: www.usp.org/worldwide/dqi/resources/technicalReports. Accessed 11 October 2008.

23. Dondorp AM, P N Newton, M Mayxay, W Van Damme, F M Smithuis, et al. (2004) Fake antimalarials in Southeast Asia are a major impediment to malaria control: multinational cross-sectional survey on the prevalence of fake antimalarials. Trop Med Int Hlth 9: 1241-1246.

24. Lon CT, Tsuyuoka R, Phanouvong S, Nivanna N, Socheat D, et al. (2006) Counterfeit and substandard antimalarial drugs in Cambodia. Trans R Soc Trop Med Hyg 100: 1019-1024.

25. Basco LK (2004) Molecular epidemiology of malaria in Cameroon. XIX. Quality of antimalarial drugs used for self-medication. Am J Trop Med Hyg 70: 245–50.

26. Atemnkeng MA, De Cock K, Plaizier-Vercammen, J (2007) Quality control of active ingredients in artemisinin-derivative antimalarials within Kenya and DR Congo. Trop Med Int Hlth 12: 68-74.

27. Anon (2007) Survey of the quality of antiretroviral medicines circulating in selected African countries. Available: http://www.who.int/entity/medicines/publications/ARV_survey.pdf. Accessed 29 January 2008.

28. Moher D, Schulz KF, Altman DG (2001) The CONSORT statement: revised recommendations for improving the quality of reports of parallel-group randomised trials. Lancet 357: 1191-4.

29. EQUATOR Network (2008) Available: http://www.equator-network.org/. Accessed 27 May 2008.

30. Taylor RB, Shakoor O, Behrens RH, Everard M, Low AS, et al. (2001) Pharmacopoeial quality of drugs supplied by Nigerian pharmacies. Lancet 357: 1933–1936.

31. SEARPharm Forum and WHO (SEARO) (2007) Extent of Spurious (counterfeit) medicines in India. Available: http://www.searpharmforum.org/LinkFiles/Project_and_activities_CFM_Report.pdf. Accessed 14 September 2008.

32. World Health Organization (2006) WHO guidelines for the treatment of malaria. Geneva, WHO.

33. Amin AA, Snow RW, Kokwaro GO (2005) The quality of sulphadoxine-pyrimethamine and amodiaquine products in the Kenyan retail sector. J Clin Pharm Ther 30: 559-65.

34. Amin AA, Snow RW (2005) Brands, costs and registration status of antimalarial drugs in the Kenyan retail sector. Malar J 26: 36.

35. Kaur H, Goodman C, Thompson E, Thompson KA, Masanja MI, et al. (2008) A Nationwide Survey of the Quality of Antimalarials in Retail Outlets in Tanzania. PLoS One 3:10.

36. Sengaloundeth S, Green MD, Fernández FM, Manolin O, Phommavong K, Insixiengmay V, Hampton CY, Nyadong L, Mildenhall D, Khounsaknalath L, Syhakhang L, Newton PN (in prep.) A stratified random survey of the proportion of poor quality oral artesunate sold at pharmacies in the Lao PDR – implications for therapeutic failure and drug resistance.

37. Sans B (1999) Return to Sans Serriffe. Guardian Unlimited 1st April 1999. Available at:http://www.guardian.co.uk/g2/story/0,,308487,00.html#article_continue. Accessed 18 January 2008.

38. Machin D, Campbell MJ, Fayers PM, Pinol APY (1997) Sample size tables for clinical studies. 2nd ed. Blackwell Science, Oxford, UK.

39. Reinke WA (1991) Applicability of industrial sampling techniques to epidemiological investigations: examination of an under-utilized resource. Am J Epidemiology 134: 1222-32.

40. Lemeshow S, Taber S (1991) Lot quality assurance sampling: single- and double-sampling plans. World Health Stat Q 44: 115-32.

41. Myatt M, Limburg H, Minassian D, Katyola D (2003) Field trial of the applicability of lot quality assurance sampling survey method for rapid assessment of prevalence of active trachoma. Bull World Health Organ 81: 877–885.

42. SampleLQ (2008). Available: http://downloads.biohealthmatics.com/downloads/0000000088.aspx. Accessed 26 January 2008.

43. Lanata CF, Black RE (1991) Lot quality assurance sampling techniques in health surveys in developing countries: Advantages and current constraints. World Health Statistics Quarterly 44: 133–139.

44. Robertson SE, Anker M, Roisin AJ, Macklai N, Engstrom K, LaForce FM (1997) The lot quality technique: A global review of applications in the assessment of health services and disease. World Health Statistics Quarterly 50: 199–209.

45. Valadez JJ, Weiss W, Leburg C, Davis R (2003) Assessing Community Health Programs - A Trainer's Guide Using LQAS for Baseline Surveys and Regular Monitoring. Teaching-aids At Low Cost (TALC), London. Available: http://www.coregroup.org/working_groups/Intro.pdf. Accessed 19 February 2009.

46. Morris M (2002) Pharmacist gets 30 years for diluting medicines. Kansas City Star December 6 2002, Sect A: 1.

47. Madden JM, Quick JD, Ross-Degnan D, Kafle KK (1997) Undercover care seekers: simulated clients in the study of health provider behavior in developing countries. Soc Sci Med 45: 1465-82.

48. Francis PA (2003) The Tip of an Iceberg. Pharmabiz (Mumbai): May 15th 2003.

49. IMPACT/WHO (2007) A guide to counterfeit pharmaceutical crime investigation. Available: www.who.int/entity/impact/events/Aguidetocounterfeitpharmaceuticalcrimeinvestigation.pdf. Accessed 26 January 2008.

50. Fernández FM, Cody RB, Green MD, Hampton CY, McGready R, et al. (2006) Characterization of Solid Counterfeit Drug Samples by Desorption Electrospray Ionization and Direct-analysis-in-real-time Coupled to Time-of-flight Mass Spectrometry. ChemMedChem 1: 702-705.

51. Hall KA, Newton PN, Green MD, De Veij M, Vandenaabele P, et al. (2006) Characterization Of Counterfeit Artesunate Antimalarial Tablets From Southeast Asia. Am J Trop Med Hyg 75: 804-811.

52. British Pharmacopoeia Commission (2008) British Pharmacopoeia 2008 Her Majesty's Stationery Office, London.

53. United States Pharmacopoeia (2002) USP26 - NF20. US Pharmacopoeia Convention, Inc; 3rd Ed edition (Nov 2002).

54. World Health Organization. (2006) The International Pharmacopoeia. 4th edition. Available: http://www.who.int/medicines/publications/pharmacopoeia/overview/en/index.html. Accessed 6 April 2008.

55. Dowell FE, Maghirang EB, Fernandez FM, Newton PN, Green MD (2008) Detecting Counterfeit Antimalarial Tablets by Near-Infrared Spectroscopy. J Pharm Biomedical Anal 48: 1011–1014.

56. Ricci C, Nyadong L, Felicia Yang F, Fernandez FM, Brown CD, et al. (2008) Assessment of hand-held Raman instrumentation for in situ screening for potentially counterfeit artesunate antimalarial tablets by FT-Raman spectroscopy and direct ionization mass spectrometry. Anal Chimica Acta 387: 551-9.

57. Green MD (2007) Simple, low-cost methods to rapidly identify counterfeit drugs. Symposium 116 'Poor Quality Antimalarials – Deaths, Detection and Developments', ASTMH, Philadelphia 7 November 2007.

58. ACTwatch (2008). Accessible: http://www.actwatch.info/about/program.asp. Accessed 25 May 2008.

59. Anon (2007) Duo-cotecxin and Cotecxin Public Alert. Pharmacy and Poisons Board, Ministry of Health, Government of Kenya. Available: http://www.pharmacyboardkenya.org/index.php?id=35. Accessed 10 February 2008.

60. Satchwell G (2004) A Sick Business. Counterfeit medicines and organised crime. The Stockholm Network. Available: www.stockholm-network.org/downloads/publications/2b74e489-Sick%20Business.pdf. Accessed 26 January 2008

61. Aldhous P (2005) Murder by medicine. Nature 434: 132–136.

62. Laing R, Vrakking H, Fourie B (2004) Quality and stability of TB medicines: let the buyer beware! Int J Tuberc Lung Dis 8: 1043–1044.

Box 1. Definitions

A counterfeit medicine is "deliberately and fraudulently mislabelled with respect to identity and/or source. Counterfeiting may include products with the correct ingredients or with the wrong ingredients, without active ingredients, with insufficient active ingredient or with fake packaging" [16].

Substandard medicines "Substandard medicines are genuine medicines produced by legitimate manufacturers that do not meet the quality specifications that the producer says they meet. For example, they may contain less (or more) active ingredient than written on the package. This may not be an intention to cheat, but may be due to problems with the manufacturing process." [17].

Degraded medicines may result from exposure of good-quality medicines to light, heat, and humidity. It can be difficult to distinguish degraded medicines from those that left the factory as substandard, but the distinction is important as the causes and remedies are different [18].

In addition, medicines used past their expiry date should also be regarded as poor quality–as they may also be degraded. However, there are very few data on what the expiry date for medicines used in the tropics should be, rather than the conventional 3 years. More investigation is required – three years may well be too short, or too long, for

some medicines. If medicines can be used for longer after the conventional expiry date this would have important economic and drug safety benefits.

In many reports it is unclear whether a poor-quality medicine is counterfeit, substandard, or degraded.

Box 2. Methods

We searched the medical literature through PubMed, GoogleScholar and the World Health Organization website using the keywords ‘counterfeit’, substandard’, ‘fake’, ‘medicine quality’ and ‘drug quality’ for for information and guidance related to the conduct and reporting of medicines quality surveys. PNN, FMF, and MDG created a draft document summarising the literature, and PNN, SJL, LJW, and NJW contributed to the statistical section. We then undertook a consultation by circulating multiple sequential drafts (about six) to an additional ten people who had recently published on the subject. They were contacted by e-mail and asked if they would be able to contribute—none declined. PNN incorporated their comments into this consensus document, and all participated with the iterative process and agree with the document presented here. We also posted the draft document paper on the Enhancing the QUAlity and Transparency Of health Research (EQUATOR) network web site [29] for four weeks to request comments by e-mail from a wider community and incorporated the response received.

Example

There is interest in determining the prevalence of outlets selling poor-quality co-artemether, the national first line recommended treatment for malaria, on an island called San Serriffe [37].

Random sampling

We can estimate a sample size assuming a prevalence of 50% (or p=0.5). This choice of estimated prevalence will give us the most conservative (i.e. largest) sample size needed. To determine the actual prevalence of outlets selling counterfeits with a precision of 5% (below 0.05x 2 =0.1) with 95% confidence intervals (z=1.96), we would need a random sample size (N) of ~390 (Table 6.1; $N = 4p(1-p)z^2/precision^2 = 4x0.5(1-0.5x(1.96)^2/(0.1)^2$ [38]). This means that purchases from 390 different outlets selling co-artemether would be required to obtain an objective estimate of the prevalence of those selling poor-quality co-artemether at one time point in one region.

LQAS

In LQAS sampling we set our upper threshold to 95% and the lower threshold to 80%. This means that it is acceptable for 95% of outlets selling artemether-lumefantrine (the unit) in one district (the lot) in Sans Seriffe to have good-quality medicines and unacceptable for <80% to have good quality medicines. Then we set the Type I error to 0.05 (i.e., there is a 5 in 100 chance that a district with 80% or fewer of the outlets selling good-quality drugs will go undetected) and the Type II risk to 0.10 (i.e. there is a 10:100

chance that we will inappropriately direct resources to a district in which the 95% or more of the outlets are in fact selling good-quality drugs) then our sample size would be 38 randomly selected outlets and the district would be considered unacceptable if more than 4 outlets had poor-quality artemether-lumefantrine (calculated using SampleLQ [42]). In other words, the null hypothesis that the district has at least 80% of its outlets selling good artemether-lumefantrine would be rejected if more than 4/38 outlets sold poor-quality artemether-lumefantrine.

Table 1. MEDQUARG checklist of items that we suggest should be addressed in reports of surveys of medicine quality.

Section and topic	Item	Description
**Title/abstract/keywords**	1	Identify the article as a study of medicine quality (recommended MeSH headings 'medicine quality, substandard, degraded, counterfeit')<br>Provide an abstract of what was done and what was found, describing the main survey methods and chemical analysis techniques used
**Introduction**	2	Summarise previous relevant drug quality information and describe the drug regulatory environment<br>State specific objectives
**Methods**		
Survey details	3	The timing and location of the survey; when samples collected and when samples analysed
Definitions	4	The definitions of counterfeit, substandard and degraded medicines used
Outlets	5	The type, including indices of size (e.g. turnover), of drug outlets sampled
Sampling design	6	Sampling design and sample size calculation<br>Type and number of dosage units purchased/outlet.
Samplers	7	Who carried out the sampling and in what guise. What did the collector say in buying the medicines? Definition of sampling frame. Question of interest, assumptions, sampling method(s) (including method of randomisation if random sampling used)
Statistical methods	8	Describe the data analysis techniques used.
Ethical issues	9	Whether ethical approval sought and whether the study encountered any ethical issues
Packaging	10	Packaging examination and reference standards
Chemical analysis	11	Chemical analysis and dissolution testing SOPs and location(s) of laboratory. Description of validation and reference standards used
Method validation	12	Details of laboratory method validation results, including but not limited to: Certificate of Analysis (COA) for reference standard, within and between run repeatability (RSD% for n=5-8), detection and quantitation limits, accuracy observed for reference samples, linear range for all analytes, sample preparation recovery studies, selectivity. Possibly, validation against a reference method or inter-laboratory study.

Blinding	13	Whether chemistry was performed blinded to packaging and vice versa
**Results**		
Outlets	14	The details of the outlets actually sampled, 'class' of pharmacy (e.g. public, private for profit, private not for profit, informal, itinerant)
Missing samples	15	The reasons why any outlets chosen for sampling did not furnish a sample. Do these outlets differ systematically from those in which samples were obtained?
Packaging and chemistry results	16	Packaging and chemistry results and their relationship<br>Details of products sampled - how many, in what drug classes, countries of origin, batch numbers, manufacture and expiry dates<br>Results for each analysis – packaging, % AI, dissolution<br>Additional information could be included in Supplementary Material
Category of poor-quality medicine	17	A clear statement for each medicine sample detected whether the investigators class it as genuine, counterfeit, substandard or degraded with an explanation as to why and whether the medicine was registered with the Government in the location(s) sampled
State company and address as given on packaging	18	If the names of companies and addresses not given – to give a reason as to why this information is not provided.
Sharing data with MRA	19	Whether the data shared with the appropriate MRA and IMPACT
Dissemination	20	Description of any non-covert packaging features that would allow others to detect counterfeit medicines. If publication is not possible, to consider disseminating via web-based Supplementary Material
**Discussion**		
Key results	21	Summarise key results with reference to study objectives
Limitations	22	Discussion of limitations of study, especially how robust the estimates of prevalence are and how applicable they may be to wider geographical areas. Discuss the direction and extent of any potential bias
Interpretation	23	An interpretation of the results, in conjunction, with prior studies, in relation to public health
Intervention	24	Whether interventions are thought appropriate and, if so, what type

**Other Information**		
Conflict of interest	25	State any potential conflicts of interest
Funding	26	Give the source of funding and role of funders in the study
Supplementary information	27	If the journal allows, suggest to list important analytical methods and additional results which would allow others to replicate the work and compare with the reported study

RSD, relative standard deviation; SOP, standard operating procedure; AI, active pharmaceutical ingredient; IMPACT, International Medical Products Anti-Counterfeiting Taskforce

Appendices

Appendix 1. Dissolution testing

The process by which the active pharmaceutical ingredients in a pharmaceutical preparation enter into solution is referred to as dissolution and in-vivo this may be a significant contributor to the medicine's bioavailability. Factors such as tablet hardness, type of excipients and physico-chemical properties of the medicine, such as particle size, crystallinity and aqueous solubility, may affect the dissolution rate, which should be tested as part of a quality survey. The presence of incorrect excipients as well as poor manufacturing processes may contribute to poor dissolution resulting in lower bioavailability. Also poor storage conditions resulting in decomposition products may influence dissolution. Even if the amounts of active pharmaceutical ingredients in a medicine are within specified limits, the amounts actually released may be lower because of poor dissolution characteristics. The dissolution rate also relies on the efficient disintegration of the dosage form and noncompliance with disintegration testing methods may quickly indicate a poor-quality medicine. Testing criteria and compliance limits for dissolution and disintegration for most medicines are described in pharmacopoeias. The difficulty facing laboratories of the considerable cost of obtaining reference standard chemicals for calibration of equipment could be addressed by repositories and a network of laboratories, and facilitated by the main manufacturers.

Appendix 2. Summary of common chemical analysis techniques to investigate medicine quality and their respective advantages and disadvantages.

(A) Common hyphenated laboratory methods of analysis

Separation Technique	Pros	Cons	Detection Technique	Type of Detector	Pros	Cons
Gas Chromatography (GC)	Fast. Good for organic volatiles. Allows headspace analysis of solvents in blisterpack.	Polar drugs may need derivatization. Labile drugs may decompose in high temperature oven.	Point detector (FID, TCD etc.)	Flame Ionization detector (FID)	Wide dynamic range, good sensitivity to carbon-containing compounds.	Requires several types of compressed gases. Does not provide drug identity.
				Thermal conductivity detector	Universal	Less sensitive than above, also requires gases
			Mass spectrometric detector	Quadrupole	Simple, offers approximate molecular weight of unknowns	Medium complexity and maintenance. Structural information provided is limited
				Ion Trap	As above plus possibility of several stages of MS (MS^n)	Medium complexity and maintenance. Medium dynamic range
				Time-of-flight (TOF)	Rapid. Provides accurate mass information for identification	More expensive than quadrupoles and ion traps.
High Pressure Liquid Chromatography (LC) (also referred to as HPLC)	One of the most widespread pharmaceutical analysis techniques	Requires solvents, expensive columns, and trained personnel. Cost depends on detector used	UV-Vis	Fixed or a few wavelengths	Simple and sensitive	Provides no or limited peak purity information
			Diode Array		Can identify compounds by spectrum database comparison. Offers peak purity assessment	More expensive than fixed wavelength detectors.
			Mass Spectrometric (see also MS detectors described for GC above)	Triple quadrupole	Preferred tool for selective drug quantification	Most models do not provide accurate mass
				Hybrid quadrupole TOF or quadrupole-ion trap	Maximum structural information. Useful for biologicals	Expensive and steep learning curve
			Refractive Index	------	Universal. Useful for drugs not absorbing light.	Less sensitive than absorciometric. Affected by temperature

			Fluorometric	------	Very sensitive and selective	More costly than absorciometric. May require derivatization
			Electrochemical (e.c.d)		Highly specific and sensitive	Costly to purchase. Some analyses require derivatisation and need a level of experience to operate in the reduced mode (for artemisinin derivatives)
Capillary Electrophoresis (CE)	Can separate acidic, neutral and basic drugs in single run. Higher resolution than LC. Useful for biologicals. Chiral analysis simpler than with LC.	Instrumentation not so widely available. Coupling to MS difficult. Less robust than HPLC. Needs high voltage. Limited to small injection volumes.	Fixed wavelength or Diode Array	------	Similar to LC	Similar to LC

(B) Laboratory methods that require no chemical separation

Technique	Type	Pros	Cons
Direct Ionization mass spectrometry (also referred to as Ambient MS)	Desorption Electrospray Ionization (DESI)	Non-destructive. Rapid. Identification and quantitation of drugs and excipients in large mass range.	Medium complexity instrumentation. Requires MS detector.
	Direct Analysis in real Time (DART)	As above. Simple spectra. Better suited for small drug molecules.	As above.
Matrix-assisted Laser Desorption Ionization (MALDI) mass spectrometry	Single stage TOF	Allows determination of molecular weight and purity of biological pharmaceuticals. Fast.	Not widespread. Sophisticated and costly instrumentation.
	Tandem TOF	As above plus possibility of quantification and determination of protein sequence	Not widespread. Sophisticated and costly instrumentation.
Inductively coupled plasma spectrometry (ICP)	Optical emission	Provides elemental composition of drug formulation. Large dynamic range	Expensive. High maintenance cost. Requires constant supply of high purity gases.
	Mass spectrometric	Provides elemental composition, very sensitive for detection of trace	As above.

		impurities. More selective than optical.	
Nuclear magnetic resonance spectroscopy (NMR)	Various	Many experiments possible with one instrument	Quantitation difficult<br>Moderate sensitivity, requires plenty of sample. Destructive (requires dissolution). High cost.
X-ray diffraction (XRD)	-------	Enables identification of inorganic and organic excipients, and crystallinity	Extremely high cost, skilled operator required. Not all organic components may be detected.

(C) Field methods of analysis

Method	Pros	Cons
Colorimetric tests	Very simple and specific<br>Can be quantitative with portable photometer	Different drugs require different tests and chemicals.
Thin layer chromatography (TLC)	Simple and moderately specific<br>Drug identity can be verified if standards are available<br>Semiquantitative	Requires some operator skills<br>Organic solvents needed<br>Cannot identify unknowns
Refractometry	Quantitative	Non-specific
Near infrared spectroscopy (NIR)[b]	Can identify unknown AIs and verify quality<br>In many cases, may work through packaging	Requires relatively pure sample<br>Costly. Not quantitative
Raman spectroscopy[b]	Can identify unknown AIs and verify quality<br>In many cases, may work through packaging<br>Very sensitive<br>Portable	Requires relatively pure sample<br>Excipients may fluoresce, making measurements difficult<br>Costly, but less expensive than NIR
Ion mobility spectrometry (Differential Mobility Spectrometry (DMS) or Drift Tube Ion Mobility Spectrometry (DTIMS))	No vacuum needed, low maintenance, compact.<br>Detection principle orthogonal to optical methods (NIR, Raman). Widely used by law enforcement agencies and airport security.	Medium resolution and intermediate cost, identity is verified by comparison with standards
X-ray fluorescence (XRF)	Enables field measurement of elemental composition<br>Semiquantitative. Can identify salt form (Br, Cl, etc.)	Costly

[b]Besides fieldable instrumentation, more sophisticated spectrometers can be used in laboratory settings.

Appendix 3. Analytical Method Validation

Common to all analysis methods is the crucial need for validation, which is the culmination of the method development process, to demonstrate that the method meets the required quality standards to robustly answer the survey questions, and that the method performance is consistent over the duration of the analysis stage of the survey (ICH 1995). Method validation consists of "documenting the quality of an analytical procedure, by establishing adequate requirements for performance criteria, such as accuracy, precision, detection limits, etc. and by measuring the values of these criteria" (Taylor and Opperman 1988). Analytical methods should be documented as openly accessible standard operating procedures (SOPs), and proof that validation has been carried out on the chosen method is essential. Three types of method validation exist. *Full validation* consists of all the steps carried out within one laboratory to validate a method adopted from the literature or developed in house. *Partial validation* is conducted for modifications of already validated methods, which can include changes in instruments, transfer of method etc. If more than one laboratory will be involved in a medicine survey, *inter-laboratory validation* (or cross-validation) should be performed in addition to the already existing full validation. The performance criteria to be reported during method validation are related to the presence of *random errors* (precision), *systematic errors* (accuracy and bias), and detection limits (minimum quantity or concentration distinguishable from the background signal). Additional performance criteria that influence the validity of the method are the linear range, sensitivity (i.e. the quantification limit expressed as minimum amount or concentration that can be quantified), the

selectivity (the ability to separate signal form the AI and common interferences such as impurities, degradation products or co-formulated compounds). Three golden rules should be followed during method validation (Massart *et al.* 1997):

1) *Validate the whole method*, including sample storage, sample preparation, analytical determination and data analysis.
2) *Validate over the whole range of concentrations*, the estimates for many performance parameters, such as precision and bias, may be dependent on the concentration, and should be therefore validated.
3) *Validate over the whole range of matrices,* if different pharmaceutical dosage forms are tested within one survey, the methods in use should be validated for each sample type, with special attention to the effect of the sample matrix on the analyte recovery and selectivity if different co-formulations are examined.

References

ICH (1995) Guideline for Industry Text on Validation of Analytical Procedures ICH-Q2A. Available: www.fda.gov/cder/Guidance/ichq2a.pdf. Accessed 11 October 2008.

Massart DL, Vandeginste BGM, Buydens LMC, de Jong S, Lewi PJ, Smeyers-Verbeke J (1997) Handbook of Chemometrics and Qualimetrics: Part A. Elsevier, Amsterdam.

Taylor JK, Opperman HV (1988) Handbook for the Quality Assurance of Metrological Measurements, Lewis Publishers.
